# Challenges in Polyglutamine Diseases: From Dysfunctional Neuronal Circuitries to Neuron-Specific CAG Repeat Instability

**DOI:** 10.3390/ijms26199755

**Published:** 2025-10-07

**Authors:** Roxana Deleanu

**Affiliations:** Institute for Neuroanatomy, Medical University of Innsbruck, 6020 Innsbruck, Austria; irina-roxana.deleanu@i-med.ac.at

**Keywords:** neurodegenerative genetic diseases, movement disorders, movement coordination circuitry, cell-type specific vulnerability, tandem-repeat expansion, single-nucleus transcriptomics, genetic instability, neuronal mosaicism

## Abstract

Several genetic diseases affecting the human nervous system are incurable and insufficiently understood. Among them, nine rare diseases form the polyglutamine (polyQ) family: Huntington’s disease (HD), spinocerebellar ataxia types 1, 2, 3, 6, 7, and 17, dentatorubral pallidoluysian atrophy, and spinal and bulbar muscular atrophy. In most patients, these diseases progress over decades to cause severe movement incoordination and neurodegeneration. Although their inherited genes with tandem-repeat elongations and the encoded polyQ-containing proteins have been extensively studied, the neuronal-type-specific pathologies and their long pre-symptomatic latency await further investigations. However, recent advances in detecting the single-nucleus transcriptome, alongside the length of tandem repeats in HD post-mortem brains, have enabled the identification of very high CAG repeat sizes that trigger transcriptional dysregulation and cell death in specific projection neurons. One challenge is to better understand the complexity of movement coordination circuits, including the basal ganglia and cerebellum neurons, which are most vulnerable to the high CAG expansion in each disease. Another challenge is to detect dynamic changes in CAG repeat length and their effects in vulnerable neurons at single-cell resolution. This will offer a platform for identifying pathological events in vulnerable long projection neurons and developing targeted therapies for all tandem-repeat expansions affecting the CNS projection neurons.

## 1. Nine Human-Specific Diseases Need Better-Defined Primary Pathogenicity

### 1.1. The Polyglutamine Disease Family and the Mutated Genes

**Figure 1:**
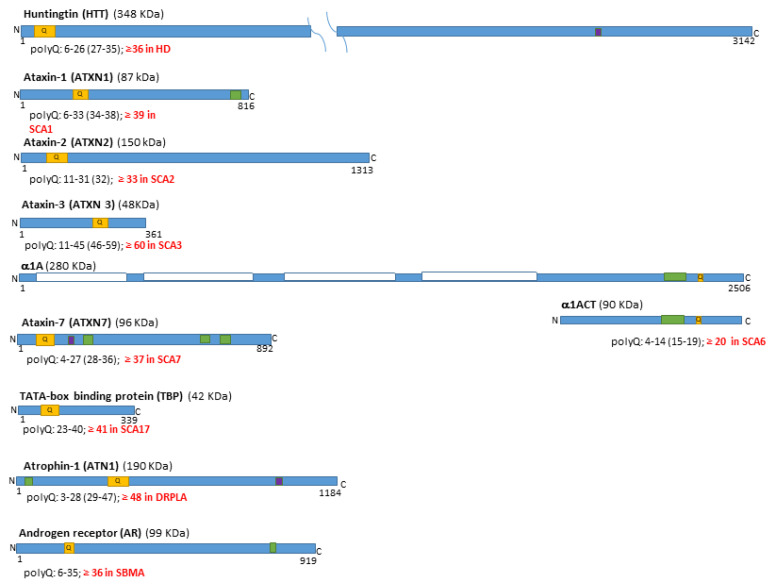
Ten human proteins containing polyglutamine (polyQ) tracts are associated with nine rare genetic diseases. These proteins have varying numbers of glutamine (Q) repeats at different positions (their location is marked in orange). When the inherited elongation of the CAG tandem repeats in the corresponding gene, and the related polyQ tract, exceed a threshold value [8] (marked in red for each gene and the encoded protein), it leads over several decades to the respective polyQ disease: Huntington’s Disease (HD), Spinocerebellar Ataxia (SCA) types 1, 2, 3, 6, 7, and 17, Dentatorubral Pallidoluysian Atrophy (DRPLA), and Spinal and Bulbar Muscular Atrophy (SBMA). The values in brackets indicate intermediate elongations [8]. Most of these proteins translocate into the nucleus, some containing sequences that act as nuclear localization signals or nuclear export signals (marked in violet and green, respectively). The only polyQ protein located within the cell membrane (domains marked with while boxes) is the longest isoform of the α1A protein; however, its gene is bicistronic and produces the nuclear protein α1ACT, which shares the same sequence as its intracellular terminal domain [5,9].

The human genome contains nine evolutionarily conserved genes (*HTT*, *ATXN1*, *ATXN2*, *ATXN3*, *CACNA1A*, *ATXN7*, *TBP*, *ATN1*, and *AR*) containing cytosine-adenine-guanine (CAG) or cytosine-adenine-adenine (CAA) triplet repeats, which encode proteins with (polyQ) tracts in their structures. The process of CAG-repeat expansion in these nine genes, both within and across generations, can lead to pathological expansions of DNA, RNA and polyQ tracts in the encoded proteins, resulting in the human-specific rare diseases known as the polyQ disease family: Huntington’s Disease (HD), Spinocerebellar Ataxia (SCA) types 1, 2, 3, 6, 7, and 17, Dentatorubral Pallidoluysian Atrophy (DRPLA), and Spinal and Bulbar Muscular Atrophy (SBMA) (reviewed in [1,2,3,4,5,6,7]; Figure 1).

Some of these genes and the proteins they encode were discovered and named after the disease caused by the expansion of their tandem repeats: the Huntingtin gene (*HTT*) and protein (HTT), mutated in HD; the Ataxin genes (*ATXN1*, *ATXN2*, *ATXN3* and *ATXN7*) and proteins (ATXN1, ATXN2, ATXN3 and ATXN7), mutated in SCA1, 2, 3, and 7 respectively; and the Atrophin 1 gene (*ATN1*) and protein (ATN1), mutated in DRPLA. For the other two polyQ SCAs (SCA6 and SCA17) and SBMA, the names of the mutated genes derive from their encoded protein‘s function or structure: the *CACNA1A* gene mutated in SCA6 is bicistronic, encoding two proteins, the first being the main subunit of the calcium channel Cav2.1, called the protein α1A, and the second a small nuclear protein, named based on its structure, α1ACT (representing the C-terminal fragment of α1A); the *TBP* gene mutated in SCA17 encodes the TATA-box binding protein (TBP), while the *AR* gene mutated in SBMA encodes the androgen receptor (AR) protein.

In most polyQ disease patients, the tandem repeat is expanded in only one allele, the disease manifesting in an autosomal dominant manner; however, rare homozygous patients, described in HD, SCA1, SCA3, SCA6, SCA17, and DRPLA, exhibit earlier onset, faster disease progression, and more severe symptoms than heterozygotes [10,11,12,13]. The nine polyQ diseases share not only the tandem-repeat mutation but also certain clinical and neurodegenerative features. However, the severity of symptoms and progression of brain pathologies vary considerably between patients, even those with similar gene mutations. While most polyQ disease symptoms onset in adults, rare cases of juvenile or even infantile forms have been documented, which exhibit longer inherited repeats and faster disease progression [1,3,4,5,6,7,14,15,16,17,18,19,20]. Interestingly, relatively similar clinical aspects and neurodegenerative features are due to CAG repeat elongations in the non-coding regions of different genes, such as the *PPP2R2B* gene in SCA12 [1,6].

### 1.2. Dysfunction and Degeneration of the Motor Coordination Network Stations

**Figure 2:**
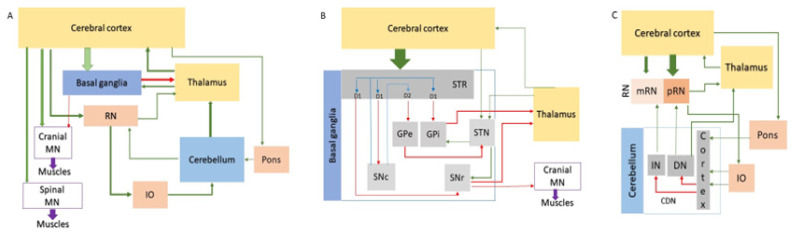
The main stations and pathways in the human motor coordination network. (**A**) Oversimplified box-and–arrows diagram of the cortico-basal ganglia and cortico-cerebellar network. (**B**) The cortico-basal ganglia pathways. The striatum (STR) contains neurons that project to the globus pallidus externus (GPe), globus pallidus internus (GPi), substantia nigra pars reticulata (SNr), and pars compacta (SNc). The neurons in SNr and GPi send the output of the basal ganglia to the thalamus. The striatal neurons that project to SNr/GPi express D1 receptors for dopamine, while those that project to GPe express D2 receptors. (**C**) The cortico-cerebellar pathways. The connection stations between the cerebral cortex and the cerebellum include several paired nuclei: the red nuclei (RN), inferior olives (IO), and pontine and thalamic nuclei. The projections from the cerebral cortex reach the neurons situated in the parvocellular and magnocellular parts of RN (pRN and mRN), which send signals to the cerebellar cortex via the neurons located in the IO. The output of the cerebellar cortex targets the cerebellar deep nuclei (CDN), including the dentate nucleus (DN) and the interposed nucleus (IN). From them, excitatory projections extend to the thalamus, directly or via the RN, and further to the cerebral cortex. Excitatory pathways are shown in green, inhibitory pathways in red, and the dopaminergic pathway in blue [21,22].

All polyQ patients develop symptoms related to uncontrollable movements (such as chorea, ataxia, and saccades), along with specific symptoms that vary depending on the affected gene and the disease stage. These symptoms are associated with dysfunction and degeneration in different central nervous system (CNS) regions, including at least one station of the motor coordination network. This comprises multiple layers and follows a hierarchical control principle, which is highly conserved among mammals. In this network, the cerebral cortex sends outputs to the effector motor neurons in the brainstem and spinal cord (which project their axons to effector muscles) after integrating input from various regulatory units, received mainly via the thalamus. The central regulatory units, which include the basal ganglia and cerebellum, receive direct or indirect input from the cortex. The cerebellum receives them mainly via the brainstem integration nuclei (such as the red nuclei (RN), located in the midbrain, pons nuclei, and inferior olives (IO), located in the medulla oblongata (MO)) and projects direct or indirect outputs to the thalamus (reviewed in [21,22]; Figure 2).

The accumulated knowledge on the connectivity and heterogeneity of brain areas responsible for motor coordination provides new directions for understanding the dynamics of polyQ pathology. PolyQ diseases present both common and specific symptomatic and anatomopathological features, some of which are reflected in their names, such as ataxia with spinal cord and cerebellar atrophy in SCAs, atrophy of the cerebellar dentate nuclei, RN, globi pallidi (GP), and subthalamic nuclei (STN, also known as corpi Luysii) in DRPLA, and atrophy of the spinal cord, medulla oblongata (MO) (also called bulbus), and muscles in SBMA.

The overall organization and function of the human basal ganglia, cerebellum, pons, IO, and RN, along with their central interconnections, are reviewed in several publications [21,22,23,24,25,26,27]. The basal ganglia consist of a paired group of interconnected subcortical nuclei (striatum (STR), GP, substantia nigra (SN), and STN) that relay cortical motor commands to ensure control of action selection and execution (Figure 2B). Each GP contains a ‘pars externa’ (GPe) and a ‘pars interna’ (GPi). Each SN includes a ‘pars compacta’ (SNc) and a ‘pars reticulata’ (SNr). The STR and STN receive excitatory inputs from the cerebral cortex, while the GPi transmits the inhibitory output of the basal ganglia to the thalamus. A network of projection neurons extends within the basal ganglia, including inhibitory (GPe, SNr), excitatory (STN), and dopaminergic (SNc) neurons [25]. Approximately half of the projection neurons in the striatum send signals to the GPi neurons, which in turn disinhibit the thalamus, thereby exciting the motor cortex and enhancing motor activity (via the direct pathway). The other half of striatal projection neurons project to GPe neurons, which inhibit the STN neurons. Excitatory neurons of the STN project to GPi neurons, which, in turn, inhibit the projection excitatory thalamic neurons and subsequently suppress cortical neurons, thereby inhibiting movements (via the indirect pathway) [21,22]. A smaller hyper-direct pathway bypasses the STR, projecting directly from the cortex and thalamus to the STN, thereby inhibiting movements [26].

The cerebellum modulates the motor pattern generated by the cerebral cortex alongside the basal ganglia, mainly for fine-tuning. The cerebellar cortex receives excitatory inputs from the cerebral cortex via the RN, pons, and IO, which are processed by the inhibitory Purkinje neurons that output to the excitatory neurons situated in the cerebellar deep nuclei (CDN) within the white matter (dentate, interposed, and fastigial). The CDN neurons send signals to the motor cortex via the thalamus, with some through an intermediate station in the RN. RNs are large, paired structures, each with two regions: the caudal magnocellular RN (mRN) and the rostral parvocellular RN (pRN). Projections from motor cortices and the dentate nucleus target pRN, while mRN receives input from the interposed nucleus. The pRN neurons also excite the IO neurons, forming a feedforward loop. IO neurons modulate Purkinje neurons, which inhibit CDN neurons and further modulate the input to the cortex, including the RN and thalamus (Figure 2C; refs. [21,24,27]).

The early stages of any polyQ disease primarily affect parts of the motor coordination network, especially the basal ganglia and cerebellum. Later stages further impact these areas, as well as other CNS regions, such as the neocortex, thalami, retinae, RN, pons, IO, and motor nuclei in the brainstem or spinal cord, with both common and variable distribution patterns among polyQ diseases (reviewed in [1,2,3,4,5,6,7,8,28]; Table 1).

HD begins with involuntary movements that gradually worsen, accompanied by rigidity, psychiatric symptoms, and cognitive decline. In the initial stages of the disease, adult HD patients experience mild chorea and oculomotor impairments, such as saccadic eye movements. During the middle stages, chorea becomes prominent, accompanied by dysarthria, postural and gait instability, and ataxia. In the late stages, chorea is often replaced by rigidity, dystonia, and bradykinesia. Brain imaging during disease progression shows significant atrophy in the STR and neocortex, followed by the GP and thalamus, with moderate atrophy of the brainstem and cerebellum in late clinical stages.

Postmortem investigations in all HD cases reveal bilateral, symmetrical, and extensive neuronal loss in the STR, often accompanied by astrogliosis. The cerebral cortex exhibits intense neurodegeneration, though the cerebellum may also show atrophy in the cortex and CDN. Interestingly, while disease progression occurs much earlier, massive cerebellar atrophy has been detected in infantile or juvenile forms. Degeneration of the thalamus includes regions associated with the STR and the cerebellum (i.e., the motor ventrolateral nucleus). The IO, pontine, and vestibular nuclei may also be affected (Table 1; refs. [3,4,11,13,14,29,30,31,32,33]).

The polyQ SCAs share clinical features such as ataxia, dysarthria, and ocular disturbances, but also possess specific characteristics. Some types of SCAs exhibit many symptoms like HD, mainly SCA3 and SCA17, and to a lesser extent, SCA1 and SCA2. Brain imaging reveals moderate to severe atrophy of the cerebellum and brainstem nuclei. While the cerebellum is the most affected region, other areas involved in motor coordination may also show atrophic changes. The cerebral cortex is generally unaffected in the early stages of most SCAs, the motor cortex becoming affected in the late stages of SCA1, SCA6, and SCA17 (Table 1; reviewed in [7]).

SCA1 usually begins with ataxia, speech difficulties, spasticity, and abnormal eye movements. In later stages, approximately 10 to 30 years after onset, patients develop muscle atrophy, cognitive impairments, and medullary dysfunction, which can lead to respiratory failure. The infantile and juvenile forms progress rapidly, exhibiting severe degeneration in the cerebellar cortex and deep nuclei, IO, and motor nuclei in the brainstem and spinal cord [7].

SCA2 begins with progressive involuntary movements of the limbs and slow eye movements. The juvenile forms may present with retinopathy, seizures, dysphagia, and cognitive regression. Brain imaging may reveal a markedly small cerebellum, along with atrophy of the pontocerebellar regions, brainstem, and cerebral cortex. Postmortem examination shows profound cell loss in the cerebellar cortex [7,15,16].

SCA3 begins and progresses with ataxia, pyramidal signs, and dystonia. In addition to cerebellar atrophy, widespread atrophy is detected in GP, STN, as well as the cerebral cortex, thalamus, and cranial and spinal motor nuclei. Juvenile SCA3 patients also exhibit degeneration in the STR, SN, and pontine nuclei, with massive loss of fibers in the superior and middle cerebellar peduncles and spinocerebellar tracts [7,17].

SCA6 exhibits milder clinical signs at onset, whereas brain imaging reveals extensive cerebellar and brainstem atrophy during disease progression [7,34]. Postmortem studies reveal massive degeneration in the cerebellar cortex, but also widespread neurodegeneration, such as in SCA1, 2, 3, and 7. However, extracerebellar neuronal loss tends to be less severe than in other SCAs, and the basal ganglia are mainly spared [7]. The juvenile form of SCA6 shows severe neurodegeneration in the cerebellar cortex, dentate nucleus, and IO. Purkinje neurons are the most affected cell type in both adult and juvenile SCA6 [18].

SCA7 manifests as progressive cerebellar ataxia and loss of vision. Neurodegeneration primarily affects the cerebellum and brainstem, particularly the IO, as well as the retina, resulting in a gradual decline in visual acuity that often leads to blindness. Juvenile and infantile SCA7 show significant atrophy of the cerebrum and cerebellum, absent or reduced deep tendon reflexes, seizures, dysphagia, myoclonus, absence of cough reflex, and severe hypotonia. The most affected cell types in SCA7 are retinal, cerebellar, and medullary neurons. The patients present also with less typical symptoms for polyQ diseases, such as hepatomegaly, haemangiomas, and congestive heart failure [7,19,35,36,37].

SCA17 shares typical features of SCAs, including ataxic gait, dysarthria, spasticity, tremor, oculomotor dysfunctions, as well as symptoms with HD, such as dysphagia, chorea, dystonia, rigidity, pyramidal signs, psychiatric symptoms, and cognitive decline. Neuroimaging shows prominent cerebellar atrophy and milder cerebral atrophy. Postmortem brains show neuronal loss in the STR, ventral thalamic nuclei, cerebellar cortex, dentate nucleus, SN, and IO [38].

DRPLA exhibits symptoms associated with both HD and SCAs. Most patient manifests with choreic movements, ataxia, myoclonus, epilepsy, and dementia. Brain imaging demonstrates severe atrophy of the cerebrum and cerebellum, with mild atrophy of the brainstem and spinal cord. Postmortem brains are atrophic, exhibiting severe degeneration of the GP, STN, and dentate nuclei, moderate degeneration of the RN, and mild neuronal loss and gliosis in the cerebral cortex [39]. Patients with juvenile-onset DRPLA often experience progressive myoclonic epilepsy as one of the initial symptoms, with onset occurring in the first years of life. Disease onset can be as early as 6 months of age, when hyperkinetic and involuntary movements, difficulty controlling head movements, and seizures develop. Juvenile-onset cases tend to show more pronounced pallidoluysian (GP and STN) degeneration compared to dentatorubral (dentate nucleus and RN) degeneration, which is opposite to the pattern observed in adult onset [39].

SBMA is a form of medullary and spinal degeneration associated with muscular atrophy. Brain imaging reveals changes in the white matter of the corticospinal tracts. Neurodegeneration primarily involves the loss of motor neurons in the spinal cord and brainstem. There is a massive degeneration in the anterior horn of the spinal cord and of the brainstem motor nuclei, except for the third, fourth, and sixth cranial nerves [20,40,41].

### 1.3. Expression and Dysfunction of the polyQ-Related Genes and Proteins

Numerous investigations have sought to elucidate the mechanisms underlying polyQ diseases, which lead to both common and specific aspects of dysfunction and degeneration in the CNS and other organs. Several studies have examined the expression of polyQ-related genes and proteins in human tissue samples. The levels of normal polyQ proteins generally mirror the respective RNA expression levels [28,39,42,43]. Table 2 presents the relative RNA levels in several human non-diseased tissue samples and neural cell types, as compiled from two databases: Gene Search | HTCA and the Human Protein Atlas (proteinatlas.org).

None of the polyQ-related genes is expressed exclusively in a single tissue type; however, only *CACNA1A* and *ATN1* exhibit high expression levels in the adult brain, while the others are moderately or lowly expressed. The gene expression across adult brain regions reveals both common patterns and differences. In the neocortex, *ATN1* exhibits the highest expression, followed by *HTT* and *CACNA1A*. Expression levels in the basal ganglia, thalamus, and brainstem are generally like those in the neocortex, except for *ATN1*. The highest cerebellar expression is observed for *CACNA1A* and *ATN1*, and moderate levels for *HTT* and *ATXN2* (Table 2). Regarding neural cell types, polyQ-related genes show differential expression in neurons and glial cells across most brain regions. In the neocortex, *ATN1* is highly expressed in both neurons and astrocytes, while oligodendrocytes show low expression; *ATXN3* is highly expressed in both astrocytes and microglia but at very low levels in neurons; *HTT* is moderately expressed in neurons, astrocytes, and microglia, yet at very low levels in oligodendrocytes. *CACNA1A* is highly expressed in neurons but at very low levels in all glial cells (Table 2). *HTT* expression level is higher in neurons than in glial cells in the striatum [44].

Several studies have explored the roles of normal proteins associated with polyQ diseases (Figure 1), identifying common and specific functions for these proteins, which are mainly involved in essential cellular functions such as transcriptional, post-transcriptional, splicing, and translational regulation, as well as intracellular transport. The small proteins ATXN1, α1ACT, ATXN7, and ATN1, along with several proteolytically derived HTT fragments, move to and from the nucleus via nuclear localization signals or nuclear export signals; the even smaller ATXN3 and TBP proteins pass freely through nuclear pores and are often found in the nucleoplasm (Figure 1; reviewed in [9]). The primary functions of the polyQ-containing proteins α1A, TBP, AR, ATXN2, and HTT are more clearly defined, whereas ATXN1, ATXN3, ATXN7, ATN1, and α1ACT have complex and not yet fully elucidated functions [42,43].

When CAG repeats expand beyond a certain threshold in each gene, they may cause structural, functional, and clearance alterations in the encoded proteins [4,5,7,9,12]. However, most documented expression levels of elongated polyQ proteins are like those of their normal counterparts in both animal models and patient cells [1,3,4,5,6]. Several studies have examined the conformational changes (misfolding), dysfunctions, and misinteractions of proteins with elongated polyQ sequences, demonstrating that all of them may acquire new functions that often and progressively result in cell toxicity; some (HTT, ATXN2, ATXN3, and ATN1) act by sequestering RNA-binding proteins or other proteins, disrupting their normal functions, while others (ATXN1, ATXN2, and AR) partially lose their normal roles. A combination of these mechanisms may also be possible. Many cells expressing expanded polyQ proteins display protein aggregate inclusions, predominantly in the nucleus or perinuclear region, throughout disease progression in all polyQ disorders, but not in all polyQ patients [45,46]. Although various organelle dysregulations have been identified and linked to cellular aggregations, their role in pathology remains a subject of debate [45,46,47,48,49]. The current understanding of the molecular mechanisms behind protein condensation includes both pathological and protective responses to cellular stressors [50,51,52,53]. Both the long latency before symptoms, often attributed to biological processes with slowly accumulating toxicity or a decades-long lag phase in forming protein aggregates, and the cell-specific toxicity, which is unrelated to the amount of the elongated protein or RNA, are not fully supported by the polyQ protein-related theories [45]. Additionally, several strategies targeting and reducing polyQ aggregates have not demonstrated benefits in recent clinical trials [54,55].

Interestingly, among the more than 80 billion neurons in the human CNS [56,57], all containing CAG repeat expansions in polyQ disease patients, many express similar levels of normal and mutated proteins (as most of the patients have only one mutated allele for the respective CAG-repeat containing gene), and several have nuclear inclusions. However, only a small proportion are extensively dysfunctional and degenerate in any of the affected regions. The mechanisms of selective degeneration have been explored in various animal and cellular models over the past 30 years [49,50]. The distribution of polyQ proteins or related RNAs in human tissues and neural cell types cannot explain the high vulnerability of neurons in the basal ganglia and cerebellum to various polyQ diseases, nor the differential neurodegeneration across tissues (Table 1 and 2; refs. [1,3,4,5,6]). Although all polyQ-related genes are expressed in the brain, their expression levels are not the highest in the CNS regions vulnerable to polyQ diseases. The levels of elongated polyQ proteins tend to stay relatively stable throughout disease progression, at least until neurodegeneration begins. Many neurons in motor coordination networks show high levels of mutated proteins but do not display obvious dysfunction, while others are functionally affected, and their condition worsens progressively and substantially.

### 1.4. The Hyper-Expansion of the Repeat Elongation Is the Novel Link for the Neuronal-Specific Pathogenesis

The early observations regarding the increases in the initial length (the number of CAG repeats in the respective gene) of the inherited mutation during the disease supported the idea that expansion length influences the severity of the disease. Additionally, the expansion of the mutation in germ cells predicts and determines earlier and more severe disease onset in subsequent generations. Intragenerational expansions guided research towards a deeper understanding of the dynamic CAG elongation in various somatic cell populations [9,58]. Several studies have highlighted that the length of a CAG repeat that is not interrupted by CAA (both coding for glutamine) is directly correlated with the onset and severity of HD [59,60] and SCAs [58]. At the same time, the CAA interruptions are associated with substantial increases in the age of onset of HD in carriers [61], indicating the specificity of the CAG tandem in elongating.

The elongations of the repeats contribute to genetic instability, meaning that the inherited DNA sequence is modified during a cell’s lifetime. This also leads to somatic mosaicism, as the sequences of the inherited genes are selectively altered in different cells, even if they exhibit the same phenotype.

Although several studies have reported relatively modest CAG expansions during the patient’s disease progression in accessible cell populations, such as blood cells, the extent of elongation was significantly higher in non-dividing cells, such as in striatal neurons of HD post-mortem patient brains [44], indicating the hyper-elongation and the concomitant massive transcription dysregulation (transcriptopathy) as the primary mechanism underlying HD-related neurodegeneration. After an intense 30-year debate, a prevailing view is that the genetic instability of the CAG repeats, which ultimately led to hyper-elongated CAG tracts and transcriptopathy, is the primary contributor to cell dysfunction in tandem-repeat diseases with or without elongated proteins [1,60].

These significant new findings, along with the progress in the related research methodology to detect the length of the elongations, to define the phenotype [62,63]. and function [64] of the most affected neurons and to in vivo studies using several mouse models with hyper-elongated CAG repeats, neuronal dysfunction and neurodegeneration, are briefly overviewed in the next section, paving the way for a deeper understanding of neuronal-specific pathology in polyQ and other tandem-repeat diseases.

## 2. Approaches for Linking Neuronal-Type-Specific Vulnerability and Genetic Instability

### 2.1. The Neuronal Complexity, Excitability, and Vulnerability to Repeat Elongation in the Basal Ganglia Circuits

At the center of the basal ganglia circuits, there are various types of striatal projection neurons (SPNs), which are inhibitory (GABAergic). Due to their morphological features, SPNs are also known as medium spiny neurons (MSNs). They comprise the majority (approximately 90%) of the striatal neuronal population, with the remainder consisting of GABAergic and cholinergic interneurons [65]. An intriguing aspect of SPN function is the modulation by the dopamine secreted by the SNc projection neurons. SPNs have two main subtypes with different functions: one subtype expresses substance P and D1 dopamine receptors, which enhance excitability through dopamine, participating in the direct pathway (dSPNs); another subtype expresses enkephalin and the D2 dopamine receptors, which decrease excitability through dopamine, acting via the indirect pathway (iSPNs) (Figure 2B; refs. [25,66]). Single-nuclei transcriptomics further classified the canonical neuronal types of the human striatum, including iSPNs and dSPNs, as well as several less abundant cell types, such as a few D1/D2-hybrid projection neurons, striatal interneurons, and glial cells [50].

Interestingly, the firing rates of SPNs increase in several HD models, both in pre-symptomatic and early symptomatic stages; however, the balance shifts towards decreased rates as the disease progresses [67]. Among striatal neurons, iSPNs are the first to degenerate, both in patients [44,45,46,47,48] and in several mouse models of HD [49,50]. The following disinhibition of targeted thalamic neurons leads to hyperkinetic symptoms, including saccadic eye movements and chorea. Later, when dSPNs are also significantly affected, dyskinesia is replaced by akinesia and muscle stiffness [21]. The SPNs are the most affected in HD, but they are also substantially affected in SCA3 and SCA17, especially in the juvenile forms [7,17,38].

GP also comprises mainly inhibitory projection neurons (GPPNs), which are larger than SPNs and are approximately 100 times fewer in number, indicating a high convergence in the striatal-pallidal projection. The GPPN axons extend over long distances towards the STN and ventral thalamic nuclei. The main types of GPPNs differ in their expression of calcium-buffering proteins, which confer their ability to sustain high firing rates. Most of the GPPNs (80–90%) co-express parvalbumin and calretinin, while fewer express only calretinin or parvalbumin. The morphology and calcium-binding protein expression of GPi neurons resemble those of GPe neurons. However, the different striatal afferents are reflected in their neuropeptide expression: substance P in the GPi and enkephalin in the GPe. SNr projection neurons (SNrPN) express mainly parvalbumin and share several features with the GPi neurons. The STN contains projection excitatory neurons (STNPN), along with a minor population of inhibitory interneurons [21,22]. Interestingly, both the excitatory STNPNs and inhibitory GPPNs and SNrPNs display intrinsic pacemaker activity [68,69].

Altered firing patterns of the GPPNs, SNrPNs, and STNPNs have been observed in HD rodent models at pre-neurodegenerative stages. All these neurons are affected in later stages, paralleling the degeneration of SPNs [70,71]. STNPN also degenerated early after the beginning of the symptoms in SCA2 and SCA3 [33]. Histopathological analyses reveal massive SNPN loss in SCA17 [38]. DRPLA patients have severe degeneration of the GPPNs and STNPNs, more pronounced in juvenile-onset forms [39].

### 2.2. The Neuronal Complexity, Excitability, and Vulnerability to Repeat Elongation in the Cerebellar Circuits

The complex functions of the cortico-cerebellar circuits rely on a complex neuronal network, centered by a distinct type of inhibitory projection neurons, the Purkinje neurons [21,22,64,68,69]. Their cell bodies are situated in the middle of the cerebellar cortex, their projections making special connections with other cerebellar neurons, as well as with neurons in intermediate stations, such as the IO (Figure 2C). The dendrites of the Purkinje neurons make synapses in the external (molecular) layer of the cerebellar cortex with the numerous small granule neurons (also known as granule cells) and the fewer unipolar brush neurons (also known as unipolar brush cells), which are excitatory and have their cell bodies in the inner (granular) layer. They receive excitatory signals from the cerebral cortex via the mossy fibers, mainly with a station in the pontine nuclei (Figure 2C). Information from approximately 25 million mossy fibers is distributed to around 50 billion granule neurons and about 25 million unipolar brush neurons. Their axons form the parallel fibers that synapse with the expansive dendritic trees of Purkinje neurons at right angles; in humans, there are approximately 3000 granule neurons per Purkinje neuron. Activation of a parallel fiber produces an excitatory response in the Purkinje neurons called a ‘simple spike’. Inhibitory interneurons in the external (molecular) cortical layer also influence circuit topography by forming synapses with the dendritic trees of Purkinje neurons and regulating their activity.

Apart from receiving input via the parallel fibers, each Purkinje neuron gets extrinsic excitatory signals from neurons of the paired IO nuclei, located in the superior medulla. They have a crenated shape and include the principal olive, as well as the medial and dorsal accessory olives. IO projection neurons (IOPNs) are large, glutamatergic, with spherical dendritic trees; the dendrites of several IOPNs form the so-called ‘glomeruli’, which facilitate the electric coupling via gap junctions. Their axons form the “climbing fibers” that reach the contralateral cerebellar cortex and synapse on Purkinje neurons, creating the most powerful synaptic contact in the brain with a 1:1 ratio. As there are about ten times more Purkinje neurons than IOPNs, each IOPN generates an average of ten ‘climbing’ fibers. Activation of a climbing fiber produces an all-or-none excitatory response in the Purkinje neurons called a ‘complex spike’.

Even when isolated from all excitatory synaptic input, Purkinje neurons possess the remarkable property of firing spontaneously at extremely high frequencies and in a highly regular manner. Purkinje neurons also express high levels of calcium-buffering proteins, such as parvalbumin and calbindin, which enable them to sustain high firing rates [68,69]. The axons of the Purkinje neurons represent the sole output of the cerebellar cortex toward the CDN (from which the biggest dentate nuclei resemble the crenated shape, as the IO nuclei). Around 30 to 50 Purkinje neurons converge onto a single excitatory deep cerebellar projection neuron (DCPN). DCPNs also fire spontaneously but are also overly sensitive to changes in afferent Purkinje neuron rates. DCPN axons send projections to the different projection neurons in the RN (RNPNs), which are also long projection excitatory neurons that receive inputs from both the CDN and the cerebral cortex [68,69].

A detailed characterization of cerebellar, pontine, IO, and RN cells was recently carried out using high-throughput single-cell sequencing [63,70,71,72,73]. These high-throughput results indicate that there is still much to be learned about the composition and functions of human cerebellar networks. Despite their generally regular shape and functions, the neurons within each subclass form a heterogeneous group, with different subsets identified by various molecular markers, such as co-neurotransmitters, neuromodulators, and regional indicators.

Several studies have revealed abnormalities in the excitability of various neuronal populations in the cerebellum associated with polyQ diseases. Purkinje neurons at early time points of the SCA1 and other SCAs showed the highest enrichment in genes related to synaptic signaling. Synapses between the parallel fiber and Purkinje neurons require glutamatergic signaling, which has been previously reported to be dysfunctional in multiple cerebellar disorders [74]. The most affected cerebellar cells in both adult and juvenile SCA1, SCA2, SCA6, SCA7, and SCA17 are Purkinje neurons [18]. By contrast, in SCA3, DRPLA, and SBMA, the most affected are the projection excitatory neurons of the CDN, as well as those in the RN, STN, thalamus, and neocortex [7,17,20,39,40,41].

Again, the level of expression of the affected gene and protein cannot explain the increased vulnerability of the respective neuronal type in the most affected CNS regions (Table 1 and Table 2; refs. [28,39,42,43,44]).

### 2.3. DNA Repair and Somatic Instability in the Neurons of the Motor Coordination Network

The long projection neurons, found throughout all stations of the motor coordination network and forming the corticobasal ganglia and corticocerebellar circuits, are generated in mammals during middle embryogenesis, which corresponds to the late embryonic to early fetal period in humans [75,76]. These neurons are designed to function effectively throughout life, supporting rapid and efficient motor coordination and forming a network with an extensive number of synapses. From a functional standpoint, the high firing rate of many neurons within the motor coordination network requires high energy levels and a high level of transcription to continuously produce the necessary proteins for the extensive ion channels and synapses. At the same time, sustained neuronal excitability results in a significant cost of DNA damage over the lifespan. Nevertheless, since long projection neurons are expected to live as the organism itself, there is a substantial requirement for their lifelong maintenance of integrity. Several protective mechanisms, including neurotrophic factors and DNA repair complexes, enable them to operate efficiently throughout the individual lifespan.

In non-dividing cells such as neurons, DNA elongations or constrictions may occur due to occasional strand misalignment (mis-paired repeats) during transcription or due to transient helix destabilization. Briefly, during transcription in neurons, single-stranded DNA can form small extrahelical extrusions, especially when highly expanded repeats are present. The formation of these structures depends on various factors, including sequence motifs, their “purity,” expansion length, and possibly methylation status. Long CAG repeats can form higher-order non-canonical structures, such as R-loops (slip-outs). Once these structures form, they are recognized by DNA mismatch repair (MMR) proteins, which attempt to repair this damage; repair pathways involve nicking, excision, and resynthesis of one of the two strands. Resynthesis may lead to a length-changing mutation—an expansion or contraction, depending on which strand has been nicked and excised. Instead of repairing the damage, this mechanism can contribute to the expansion of pathogenic repeats, further genetic instability, and somatic mosaicism, which are now well-recognized features in HD and other diseases with tandem repeat elongations [44,59,77,78].

More than 30 years ago, Telenius et al. measured the level of somatic mosaicism of the expanded HD allele in adult patients with late-onset HD [79]. Larger expansions were linked to increased mosaicism. Furthermore, the degree of mosaicism was high in the basal ganglia and cerebral cortex, intermediate in the blood and liver, and low in the cerebellum. Additionally, greater instability was found in sperm compared to blood. This pioneering study, along with those that follow, provides evidence that somatic mosaicism in HD varies by tissue and correlates with neuropathology [79,80,81].

The first study reporting hyper-expansions in the HD post-mortem brain identified exceptionally long CAG-repeat tracts, up to 1000 repeats, mainly in the neocortex. The solid-phase PCR (SP-PCR) used in this study relies on amplifying dilutions of DNA from bulk tissue containing heterogeneous cell types [82]. However, a more precise correlation of CAG repeat length variability with specific cell types depended on the development of sequencing methodologies at the single-cell level. One approach involved implementing fluorescence-activated cell sorting to isolate different cell populations from post-mortem HD brains or specific brain regions (striatum, cerebral cortex) based on marker gene expression. Subsequently, the isolated cells were sequenced to determine the CAG size in *HTT* transcripts, providing matched transcriptional and genetic instability profiles at the subpopulation level. Nevertheless, this method was limited by short-read sequencing, which could detect a maximum of 110 CAG repeats [31,83].

**Figure 3:**
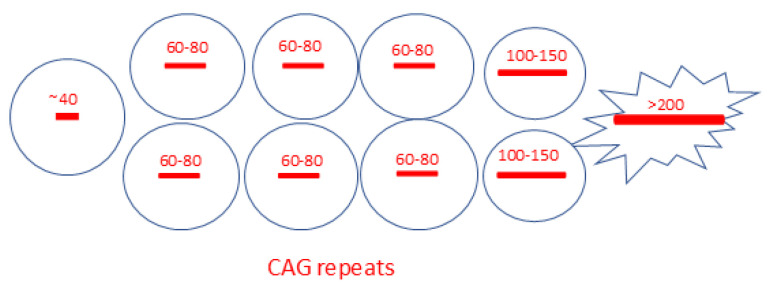
Graphical depiction of mosaicism in Huntington’s disease striatal projection neurons. The CAG repeat size in the HTT pathological allele was measured in each nucleus; their lengths ranged from a few additional CAGs to the inherited pathological allele to extreme elongations, with most CAG repeat tracts having approximately twice the original length [44].

The development of new techniques for measuring long CAG expansions at the single-neuron level [84] has significantly improved previous data and uncovered the disease’s dynamics. Handsaker and colleagues [44] advanced the technology by developing an improved single-nucleus RNA-sequencing method and an analysis based on long reads, which enables the simultaneous collection of transcriptional profiles and CAG repeat size in the *HTT* RNA from the same cell. This allowed grouping cells by both their transcriptional profile and CAG length in the *HTT* gene. Somatic CAG expansion was found to be allele-specific, mainly affecting the mutated *HTT* allele but not the other inherited allele or the alleles of unaffected individuals. While 95–98% of each HD donor’s SPNs had expanded CAG repeats with approximately 20–30 CAGs beyond the inherited length (around 40 CAGs), only a small proportion showed much longer expansions (100–500+ CAGs). A tiny proportion of SPNs exhibited extreme expansions, with more than 800 CAG repeats. The somatic mosaicism found in SPNs carrying a pathological *HTT* allele is schematically shown in Figure 3.

Remarkably, the gene expression changes in SPNs with expansions beyond 150 CAGs were highly consistent across patients, supporting a common mechanism of dysfunction and neurodegeneration. The neurons with extreme expansions were significantly dysregulated in terms of transcription, losing both their marker identity and expressing markers of cell death pathways. Striatal interneurons and glial cells showed modest CAG-repeat instability.

The altered gene expression exhibited two types of relationships to CAG-repeat length: one set of genes showed continuous changes as the CAG repeat length extended beyond 150 CAGs. In contrast, another set displayed more discrete and dramatic changes in both iSPN and dSPN with a CAG repeat length greater than 250 CAGs. The genes whose expression decreased in SPNs with a repeat length over 150 CAGs were among the most highly expressed in SPNs, suggesting that a core biological change involves eroding the SPN identity features that distinguish them from other neurons. Many of these genes also perform essential physiological functions, such as those encoding potassium channel subunits [44].

A specific set of over 100 genes, normally suppressed in SPNs, becomes active in those with 350 or more CAG repeats [44]. Conversely, SPNs showed reduced expression of genes that differentiate them from other neurons [59,85,86,87]. The set of genes activated by the hyper-expansions includes genes typically expressed in different neural cell types but not in SPNs, as well as two genes (*CDKN2A* and *CDKN2B*) that encode proteins (p16(INK4a) and p15(INK4b)) that promote senescence and apoptosis [88,89].

Interestingly, inactivation of the polycomb repressor complex 2 (PRC2) in adult mice results in similar gene expression changes, leading to SPN loss, a decline in motor function, and death within months [90]. The findings suggest that transcriptional changes in SPNs with very long repeats may contribute to their death.

The breakthrough results of Handsaker and colleagues [44] support a multi-phase model called ‘ELongATE’, in which somatic expansion rates in neurons increase significantly once they exceed 80 CAG repeats, with a threshold of approximately 150 CAG repeats needed to trigger cell-autonomous transcriptional dysregulation in SPNs, leading to even faster expansion and cell death. The asynchronicity in HD mutation elongation in SPNs occurs because length-change mutations were initially rare events (happening less than once a year per cell across 36–55 CAGs). However, once they occur, they increase the chances of subsequent elongations. The proposed ‘ELongATE’ model offers a plausible explanation for some unresolved questions. Firstly, the progressive loss of SPNs during HD neurodegeneration may result from the time needed for extreme expansion events to accumulate in these neurons over a lifetime. Secondly, the extended period of degeneration observed in HD patients—typically 10–20 years from diagnosis to death—appears to align with the rapid yet asynchronous neuronal degeneration suggested by the ‘ELongATE’ model [44]. According to this model, although all cells in the patient’s brain carry the *HTT* gene with CAG elongations, only those with more than 150 CAG repeats are truly toxic. Consequently, only a few SPNs generate highly expanded and toxic forms of DNA, RNA, and proteins, contributing to the slow, progressive degeneration of neurons. Remarkably, the expression levels of *HTT* RNA remained unchanged with CAG repeat expansion.

These findings align with analyses of a specific HD mouse model (Q175), which starts life with a CAG-repeat tract exceeding 170 CAGs in all cells [91], as well as with other studies analyzing the transcriptome in patients’ striatum at the single-cell level [92].

Future research will be necessary to ascertain whether the dynamics driven by repeat expansions in HD follow the dysfunction and neurodegeneration in other brain regions and neuronal types, or if these areas are secondarily affected due to the dysfunctions of their neuronal networks (Figure 2). Extensive expansions were previously identified in the cerebral cortex of post-mortem HD brains, as well as in mouse models [49,50]. However, the association with the most affected long projection excitatory neurons was not studied at the time due to technical limitations [31].

Interestingly, the somatic *HTT* CAG expansion was also observed in Purkinje neurons, albeit at lower levels compared to the SPNs [11,32]. However, further studies are expected to investigate the CAG elongation at the single-cell level in all the projection neurons affected by HD diseases using single-nucleus transcriptomics [44] and confirm or refute the increased expansion in other cell populations with high electrical, metabolic, and transcriptional rates, especially for those with well-developed projections and high firing rates. These include neurons in other basal ganglia nuclei (GPi, GPe, STN, and SN) as well as in various hubs of motor coordination, including the thalamus, RN, IO, and DCN. All these regions are affected in the later stages of HD (Table 1), although the underlying pathological mechanisms for these neuronal populations remain unclear.

The next challenge is to determine if and how the ‘ELongATE’ model aligns with the other eight polyQ diseases, as well as with the tandem-repeat diseases in general. The primary question concerns the ‘GATE’ for each CAG elongation for each gene, referring to the elongation level that produces massive transcriptomic dysregulation in each neuron vulnerable to dysfunction and degeneration. Can these values be close to 150 repeats for all polyQ diseases, or does the vulnerable length depend on the cell type and mutated gene?

**Figure 4:**
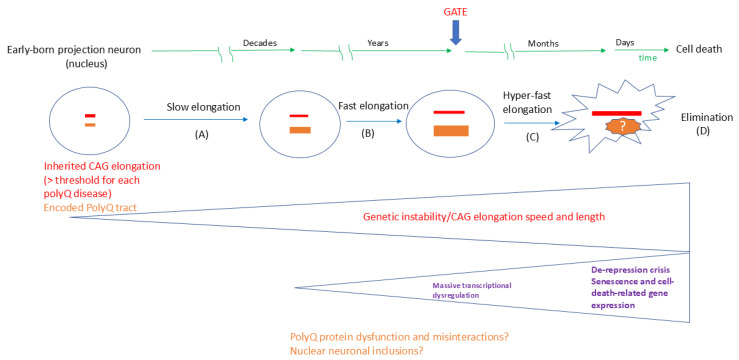
The expected pathological progression in projection neurons vulnerable to polyQ diseases. The dynamic stages of CAG expansion in Huntington’s disease (HD), along with the proposed ‘ELongATE’ model [44], could apply to other polyQ diseases. Besides CAG expansion, the model should also consider polyQ-expanded proteins in the respective nuclei. Before the CAG repeats/polyQ tracts reach the ‘GATE’ (around 150 in HD), the potential effects of protein misinteractions and transcriptomic dysregulation may be offset within neurons; however, once rapid expansion reaches the ‘GATE’, a hyper-fast elongation phase commences, accompanied by massive transcriptomic dysregulation and resulting in cell death within a relatively short period.

For polyQ diseases in general, an ‘ELongATE’ model should incorporate the elongated proteins and their potential roles in the pathological process. This includes the dysfunction and misinteractions of proteins with expanded polyQ tracts (Figure 1), as well as the dynamics and pathology of nuclear inclusions. Future research will be necessary to establish whether the dynamics of the polyQ tract correspond with CAG elongation at all stages of the ‘ELongATE’ model and whether this understanding can be extended to other polyQ diseases, different elongated genes, encoded proteins, and various vulnerable neuronal types. An extension of the ‘ELongATE’ model to all polyQ diseases, which addresses both CAG expansions and polyQ proteins, is proposed in Figure 4.

Following intensive investigations in HD, both somatic mosaicism and CAG hyper-elongations have been observed in other polyQ diseases, including several mouse models with long and hyper-elongated CAG repeats [49,50] and patient tissue samples [87].

Kacher et al. reported that the somatic instability of CAG repeats correlates with clinical progression in individuals with SCA1. Alleles of intermediate size without CAT interruptions in the *ATXN1* gene are also mutable and exhibit somatic instability. The somatic instability of both alleles in an SCA1 patient may therefore have contributed to modifying the age at disease onset, progression, or phenotypic severity [93]. The somatic instability was examined in the blood, sperm, and neuronal tissues of SCA1 patients. CAG repeats in *ATXN1* are more unstable in sperm than in blood, supporting the HD findings. Moreover, the neuronal mosaicism patterns in SCA1 resembled those in HD. The most significant instability was observed in the cerebrum, while the least was seen in the cerebellum [94,95]. Although this finding contradicts the idea that increased tissue mosaicism correlates with neuronal vulnerability, we must consider the technical limitations in detecting mosaicism in Purkinje neurons, which are a minor population in the cerebellum. The correlation between neuronal vulnerability and genetic instability has also been examined in SCA1 ‘knock-in’ mice, which carry 154 CAG repeats on one allele and two CAG repeats on the other allele. Expansions exceeding 200 repeats were observed in the striatum and spinal cord, but no expansions larger than 174 repeats were found in the cerebellum. These tissues exhibit varying levels of mosaicism, suggesting no clear correlation between mosaicism and selective neuronal vulnerability in SCA1. Moreover, repeat instability in SCA1 was found to be age-dependent. At 7 weeks, the knock-in allele remained stable; however, small expansions and contractions appeared by 30 weeks. Most larger expansions occurred after 30 weeks, following the onset of neuronal dysfunction, indicating that repeat instability relates to neuronal vulnerability [95]. However, technical limitations in addressing solely the Purkinje neurons should be considered before drawing any further conclusions.

Subsequent research uncovered similar patterns of neuronal mosaicism in SCA3 and DRPLA, despite several differences in neuropathology and neuronal vulnerabilities. The preferential expansion of the *ATXN3* CAG tract found in SPN nuclei isolated from SCA3 donors indicates that these neurons generally tend to expand into long CAG tracts. Notably, repeat expansion was observed in specific cerebellar cells in both early- and late-onset DRPLA cerebellum. These findings suggest that expansions were more common in Purkinje neurons than in granule neurons, regardless of age at onset. White matter glial cells exhibited more expansion than Purkinje neurons in late-onset tissue but showed similar levels in early-onset tissue. The study involved one early-onset case and one late-onset case; therefore, further research is necessary to draw definitive conclusions [96].

Obviously, it is essential to link the CAG elongation level with the polyQ tracts at the single-cell level using similar technology as in [44]. While the SPN, Purkinje neurons, and cortical projection neurons have already been examined for dynamic elongations, other vital cells in the motor coordination network remain unaddressed. These include at least the long projection neurons in the GPi, GPe, SNc, SNr, STN, IO, pRN, and CDN (Figure 2). Upon analyzing the genes, proteins, and most affected projection neurons of the motor coordination network in polyQ diseases, some essential pieces of the puzzle are in place, bridging breakthroughs and established knowledge; nonetheless, many remain missing, and several issues need resolution (Table 3).

Further single-cell studies are required to investigate the CAG elongation dynamics in Purkinje neurons in several polyQ diseases that predominantly affect this cell type, such as SCA2, SCA6, SCA7, and SCA17. The projection neurons in CDN, IO, pons, and RN should also be addressed in the polyQ SCAs, as well as in HD, DRPLA, and SBMA. The same is true for the long projection neurons in the basal ganglia nuclei, from which only the SPNs were addressed. The dynamic increase in CAG repeat length at single-cell resolution should be further investigated in all vulnerable neurons, as well as in the non-vulnerable neurons that comprise the motor coordination network. The information from non-vulnerable neurons could help understand the protective mechanisms in specific neurons, including DNA repair proteins, and inform the design of therapeutic approaches along these lines.

Elongated polyQ proteins and nuclear inclusions, a topic of ongoing controversy, could be addressed with novel technologies that integrate single-cell proteomics and spatial transcriptomics. While in the early stages of the ‘ELongATE’ model (A and B, Figure 3; ref. [44]), it is expected that the polyQ length will correlate with the CAG tract; however, the translation during later stages, particularly the de-repression crisis, remains uncertain. Another concern relates to the impact of polyQ length on disease progression. No significant transcriptomic changes were observed for the stages associated with slow and fast elongation in the HD striatum. However, several studies have documented HTT dysfunction and misinteractions in early disease stages, even in cellular models representing prenatal stages [1,2,3,4,5,6,7,8,49,50,97]. It remains uncertain whether proteins with polyQ elongations shorter than the ‘GATE’ level (e.g., 150 for HD) contribute to disease pathology, especially regarding DNA repair dysfunction that causes the elongations (Figure 3). The same question applies to their involvement in nuclear inclusions and their role in disease progression. Once again, various strategies aimed at targeting and reducing polyQ aggregates have not demonstrated benefits in recent clinical trials for HD [56,57].

### 2.4. Approaches to Therapeutic Targets Based on the ELongATE Model

While the goal of this review is to address the challenges related to the complexity of movement coordination circuit neurons affected by polyQ diseases, as well as to the detection of the CAG repeat lengths and their effects in vulnerable neurons at single-cell resolution, the reported therapeutic strategies based on CAG expansions will be very briefly addressed and in line with the proposed ‘ELongATE’ model. Obviously, the ultimate challenge in the polyQ disease field is to halt the pathological processes before neuronal dysfunction and degeneration occur. On this line, preventing CAG expansions from reaching the pathological level or decreasing the expansion level before the respective neuron degenerates are logical approaches. These approaches are still in the very early stages of development, limited by the fact that targeting neuronal nuclei should be done with minimal or no perturbation of their normal functions. The best approach would be to address the CAG expansion elongation as soon as possible after detecting the patient’s inherited pathological mutation; however, this is only valid if an entirely safe neuronal nuclear therapy becomes available in the future. Meanwhile, several studies have shown an effective reduction of HD symptoms and neurodegeneration after interfering with DNA repair complexes, altering the CAG repeat DNA structure, or editing the mutation in animal models [61,98,99,100,101,102,103,104,105]. The translation of these techniques to patients requires caution, and it is still in its very early stages.

**Figure 5:**
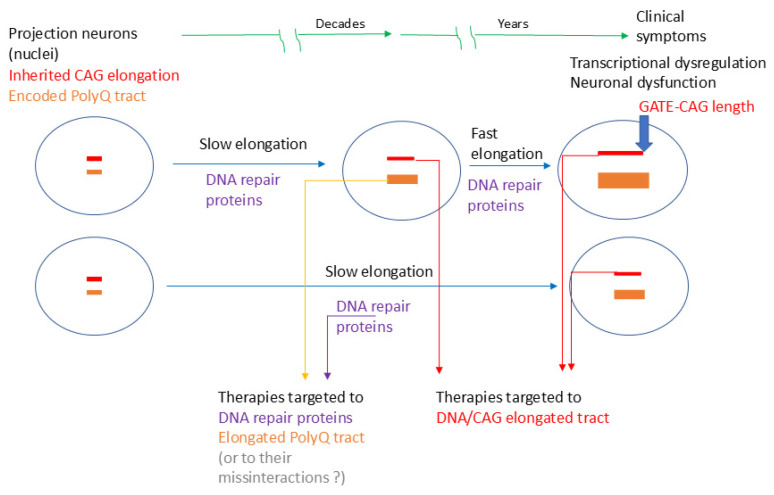
Potential therapeutic targets during the pre-degenerative stages of polyQ diseases. Therapeutic approaches should be considered before the CAG repeat expansion exceeds the ‘GATE’ threshold (of 150 CAG repeats/polyQ tract in Huntington’s disease), as illustrated in the ‘ELongATE’ model [44]. Once this threshold is surpassed, massive transcriptomic deregulation occurs, leading to cell death shortly thereafter. Future research should determine the ‘GATE’ thresholds in patient-vulnerable neurons for other polyQ diseases.

A proposed window for a therapeutic approach targeting pre-degenerative stages, aligning with disease progression as described in the ‘ELongATE’ model (Figure 4), is shown in Figure 5.

A promising strategy involves directly targeting the expanded repeat or modulating key DNA repair genes, such as by using antisense oligonucleotides or small molecules. For example, targeting the CAG repeat with a small molecule, such as naphthyridine-azaquinolone, which specifically binds to the elongated CAG repeats and induces contraction, has shown promise [101,104]. A recent successful approach involved using this molecule to target slipped-CAG repeats within the R-loop DNA structure, resulting in efficient contractions in the striatum of HD mice over a four-week treatment. Specifically, the small molecule naphthyridine-azaquinolone binds explicitly to expanded CAG repeats, causing contractions in the expanded CAG repeats within most SPNs, without affecting normal alleles or other CAG repeats in the genome. Its effect on CAG instability depends on transcription through the repeat, making it particularly effective in neurons. Extrapolating to humans affected by HD, the authors suggest that administering a drug like naphthyridine-azaquinolone before the rapid onset of somatic CAG expansions could effectively prevent expansions and promote contractions of the inherited expanded allele to shorter lengths. Treatments lasting around one year could potentially reduce the number of repeats by 5–25 repeats. For an HD allele ranging from 36 to 70 repeats, such changes could be clinically significant. Moreover, some recent novel findings in animal models of DRPLA [103] give hope for effective therapy in all polyQ diseases.

Other methods to target expanded repeats in vivo include gene editing to deactivate mutant *HTT*, which has been successful in mouse models. However, gene editing still faces challenges in patients due to issues such as brain delivery, editing efficiency, ongoing nuclease activity, off-target effects, immune responses, and haploinsufficiency. Another approach used an antisense oligonucleotide to decrease CAG expansions, but it did not induce contractions of the expanded repeat. An inhibitor of histone deacetylase 3 successfully suppressed somatic CAG expansions through an unknown mechanism. A promising strategy involves directly targeting the expanded repeat or modifying key DNA repair genes, such as by using antisense oligonucleotides or small molecules [61,104,105].

Addressing DNA repair mechanisms by modulating the expression of repair proteins, such as MSH3, could be a promising therapeutic approach. Concerning therapies targeting polyQ proteins and protein aggregates, further research is necessary to understand their roles in the disease process. Currently, the understanding of the molecular mechanisms behind protein condensation involves both harmful and protective responses to cellular stressors [50,51,52,53]. The long latency before symptom onset, often associated with the gradual accumulation of toxicity through biological processes or a decades-long delay in forming protein aggregates, as well as cell-specific toxicity, which is independent of the amount of the expanded protein or RNA, is not fully supported by polyQ protein-related theories [45]. A compelling argument for the importance of the CAG repeat as a pathological marker is that these repeats help determine relatedness in several diseases characterized by elongation in non-coding regions of their respective genes. The progression and clinical presentation of these diseases largely mirror the neuronal symptoms observed in polyQ disorders. Furthermore, various strategies aimed at targeting and reducing polyQ aggregates have not demonstrated benefits in recent clinical trials [54,55].

## 3. Conclusions and Outlook

Classical histopathological examinations of brains from patients with polyQ disease have shown that projection neurons within the motor coordination circuits are more severely affected than other neurons and cell types overall. Recent single-nucleus transcriptomic analyses of post-mortem patient brains have confirmed the significant neurodegeneration of SPNs neurons in the brains of HD patients [44]. Additionally, detecting the single-nucleus transcriptome alongside the length of tandem repeats has enabled the identification of hyperexpanded CAG repeats that induce extensive transcriptional dysregulation and activate markers of cell death in SPNs [44]. The profiles showing the most severe transcriptional dysregulation correspond to tandem repeats longer than 150 CAGs, which have been verified in several HD brains. These ground-breaking findings support the theory that hyperexpansion of repeats in long projection neurons is the key pathogenic mechanism in HD. The proposed ‘ELongATE’ model [44] offers a plausible explanation for unresolved questions in the polyQ disease field. Firstly, the progressive loss of SPNs during HD neurodegeneration may result from the time needed for extreme expansion events to accumulate in these neurons over their lifetime. Secondly, the extended period of degeneration observed in HD patients appears to align with the rapid, yet asynchronous neuronal degeneration suggested by the ‘ELongATE’ model. Consequently, only a few SPNs generate highly expanded and toxic forms of DNA, RNA, and proteins at the same time. Still, their addition contributes to the slow, progressive, and massive degeneration of similar neurons. However, several questions and challenges remain to be addressed to demonstrate the mechanisms leading to the massive transcriptional dysregulation (transcriptopathy).

The next challenge involves implementing advanced single-nucleus technologies and long-read sequencing to monitor somatic instability in all other vulnerable neuronal populations in HD, as well as in other polyQ diseases. This is crucial for verifying the extent of pathological CAG expansion in susceptible neurons and addressing the heterogeneity of neuronal populations affected in polyQ diseases.

Understanding the complexity of networks that control motor coordination in the normal human brain is essential for recognising their dysfunctions, but it also presents a considerable challenge. Considerable progress has been made recently in uncovering the cellular complexity of the human CNS, supported by technological advances in single-cell and single-nucleus RNA sequencing. Several human cell atlases and databases are now accessible to researchers, enabling the detailed characterisation of cells that are vulnerable to disease [62,63]. Beyond cell phenotype, recent functional studies have expanded understanding of the connections between neurons, as shown in the Human Connectome Project (HCP) repository [64]. Consequently, alterations at one node can propagate through the entire network, impacting functions at other nodes [23,24]. The integrated network approach provides a new perspective on the organization of basal ganglia and cerebellar circuits in relation to the cerebral cortex, as well as identifying primary and secondary pathologies in movement disorders, including polyQ diseases. This will help differentiate between cell-autonomous and non-cell-autonomous mechanisms, facilitating an understanding of disease progression. It will also support more effective monitoring of disease evolution and therapeutic responses in the near future. However, while neuronal networks may explain why different areas and cells are affected secondarily, they cannot clarify why CAG expansions in specific genes impact certain neurons.

The most affected neurons are projection neurons, characterized by high metabolic activity, elevated firing rates, and an extensive projection network. These neurons should all exhibit elevated levels of transcription and DNA repair molecules. Although inhibitory SPNs and Purkinje neurons have been functionally studied in polyQ diseases, other inhibitory and excitatory projection neurons in the basal ganglia, RN, IO, and cerebellum, all involved in movement coordination, have received less attention.

In all neurons carrying inherited CAG expansions in coding genes, CAG repeat lengths may change due to DNA misalignment during transcription, leading to the formation of small extrusions, especially when highly expanded repeats are present. Long CAG repeats can adopt higher-order non-canonical structures, such as R-loops (slip-outs). Once formed, these structures are recognized by DNA mismatch repair proteins, which attempt to repair the damage. Resynthesis may result in a length-changing mutation—either an expansion or contraction—depending on which strand has been nicked and excised. Instead of repairing the damage, this process can promote the expansion of pathogenic repeats, thereby increasing genetic instability and somatic mosaicism, features now well recognised in HD and other diseases characterized by tandem repeat expansion.

However, the level of expansions that leads to transcriptopathy remains an open question for all other projection neurons, except SPNs. The same applies to the polyQ proteins encoded by genes containing expanded and hyperexpanded CAG repeats. While their conformational changes and capacity to aggregate are well-documented, their role in CAG elongation and the development of transcriptopathy remains unclear. Conformational changes in proteins develop dynamically. Since most encoded proteins reside in the nucleus and participate in transcription, they are likely to interact with proteins involved in transcription and DNA repair. However, further research is needed to establish a direct link between their elongation and transcription, as well as DNA repair processes.

Despite notable progress in understanding the underlying mechanisms and developing potential therapies to reduce protein aggregation, the impact of these mechanisms on disease progression remains a topic of debate. Nonetheless, efforts to clarify how long expansions cause neuronal loss should continue, with new insights expected from novel human neuronal models [97] and humanized mouse models [49,50]. The same applies to therapies targeting neuronal-specific elongations and hyper-elongations. Ultimately, the main challenge is to halt the pathological process before neuronal degeneration occurs. In this context, identifying the ‘GATE’ of pathological expansion and preventing it from reaching this critical level is a logical strategy. However, approaches that require nuclear targeting in neurons need further research.

Strategies that directly target the expanded repeat or modulate key DNA-repair genes (e.g., using antisense oligonucleotides or small molecules) are among the most promising approaches for addressing this condition. As an initial step, the efficacy of these innovative therapies in delaying disease onset has already been evaluated in animal models [61,101,102,103,104,105]. While several studies have demonstrated effective reduction of HD symptoms and neurodegeneration following mutation editing or interference with DNA repair complexes in animal models [61,98,99,100,101,102,103,104,105], translating these techniques to patients remains cautious and is in its initial stages.

Nonetheless, by focusing on the primary cause of the disease and targeting relevant cells, new therapeutic strategies could slow or halt the progression of expansions. Disease-modifying therapies must include neuronal-specific studies of CAG elongations, effective methods to manipulate this process, and improved readouts for their effects, including single-cell/nucleus readouts. However, although considerable progress has been made, there are currently no clinically validated disease-modifying therapies capable of delaying, arresting, or reversing disease progression for any repeat expansion disorder. Based on the latest technological advancements [105], further collaborative efforts are anticipated in this direction.

## Figures and Tables

**Table 1:** Distribution of degeneration in the central nervous system of polyQ disease patients.

Disease	Forebrain						Midbrain			Hindbrain						SC	
	CC	TH	R	Basal Ganglia				RN	CN	Pons		Cerebellum		MO		MN	WM
				STR	GP	STN	SN			PN	CN	CBC	CDN	IO	CN		
HD																	
SCA1																	
SCA2																	
SCA3																	
SCA6																	
SCA7																	
SCA17																	
DRPLA																	
SBMA																	

PolyQ diseases affect the cerebral cortex (CC), thalamus (TH), retina (R), basal ganglia (each divided into striatum (STR), globus pallidum (GP), subthalamic nucleus (STN), and substantia nigra (SN)), red nuclei (RN) in the midbrain, cerebellum (with cerebellar cortex (CBC) and deep nuclei (CDN)) pontine nuclei (PN), inferior olives (IO) (in the medulla oblongata (MO)), cranial nerve motor nuclei (CN) (in the midbrain and hindbrain), and spinal cord (SC) with spinal motor nuclei (MN) and white matter (WM). Color intensities indicate the approximate extent of degeneration in affected areas, as described in morphopathological studies [3,5,7,28], which range from severe (brown-orange) to moderate (peach-orange), mild (creamy-peach), and mild (heather-peach), and relatively spared (white). Huntington’s Disease (HD), Spinocerebellar Ataxia (SCA) types 1, 2, 3, 6, 7, and 17, Dentatorubral Pallidoluysian Atrophy (DRPLA), and Spinal and Bulbar Muscular Atrophy (SBMA).

**Table 2:** The expression of polyQ disease-related genes in several human tissues and neural cells.

Genes	WB	CNS Regions									Neural Cell Types				H	K	B	M	S	
		NC	BG	R	TH	MB	CB	P	MO	SC	N	A	O	MG						
*HTT*																				
*ATXN1*																				
*ATXN2*																				
*ATXN3*																				
*CACNA1A*																				0–9
*ATXN7*																				10–19
*TBP*																				20–99
*ATN1*																				100–199
*AR*																				>200

The relative expression of the levels polyQ disease-related genes in different human adult normal tissues, including the whole brain (WB) and central nervous system (CNS) regions (NC-neocortex, BG-basal ganglia, TH-thalamus, R-retina, MB-midbrain, CB-cerebellum, P-pons, MO-medulla oblongata, SC-spinal cord), based on the median relative RNA level values from Gene Search | HTCA and Human Protein Atlas proteinatlas.org; the values for the neural cell types (neurons (N), astrocytes (A), oligodendrocytes (O) and microglia (MG)) correspond to the NC; H-heart, K-kidney, B-blood, M-striatal muscles, S-skin. For the sake of simplicity of the overall presentation, the relative expression levels were grouped into five intervals (very low, low, moderate, high, and very high expression) and linked to increasing color intensities.

**Table 3:** The puzzle of elongated genes and proteins in the projection neurons affected by polyQ diseases.

Disease	Most Affected Projection Neurons of the Motor Coordination Network (Patients and Animal Models)							PolyQ Proteins		CAG-Repeat Elongations Patient/Animal Models *	
	SPNs	GPPNs/ SNrPNs	STNPNs	PNs	IOPNs	DCPNs	RNPNs	DY	AG/ NI	GI	CAG Repeat Size in Neurons
HD	+++	++	+	++	+	++	+	+	+	++/+ *	>150/144 *, 150*, 175 *
SCA1	+	+	+	+++	++	++	++	+	+	++/+	?/154 *
SCA2	-	++	+	++	++	++	++	+	+	+/+ *	?/127 *
SCA3	++	++	++	++	++	+++	++	+	+	+/+ *	?/148 *
SCA6	-	-	-	+++	++	++	++	+	+	+/+ *	?/84 *
SCA7	-	+	+	++	++	++	++	+	+	+/+ *	?/266 *
SCA17	++	++	++	+++	++	+	+	+	+	+/+ *	?/105 *
DRPLA	-	+++	+++	++	+	+++	+++	+	+	++/++ *	?/129 *
SBMA	-	-	-	+	+	++	+	*+*	*+*	+/+ *	?/112 *, 113 *

The degree of the degeneration of the inhibitory (red) and excitatory (green) projection neurons in the motor coordination network, as in pathological reports [3,5,7,28], and qualitatively attributed as low (+), medium (++), high (+++), and not obviously affected (-); striatal projection neurons (STN), globus pallidus and substantia nigra pars reticulata projection neurons (GPPN/SNrPN), subthalamic nucleus projection neurons (STNPN), inferior olive projection neurons (IOPN), red nucleus projection (RNPN), deep cerebellar projection neurons (DCPNs); and Purkinje neurons (PN). Associated protein dysfunctions (DY), aggregates (AG), and nuclear inclusions were identified in several animal models and patient brains; the same is true for genetic instability (GI), characterized by an increased number of CAG repeats in the respective genes. The values marked in blue correspond to elongations that are close to the ‘GATE’ of 150 repeats proposed in the ‘ELongATE’ model [44]. The values marked with * correspond to mouse models, as in [4,45,46,51]. The question marks indicate aspects that have not been addressed. Huntington’s Disease (HD), Spinocerebellar Ataxia (SCA) types 1, 2, 3, 6, 7, and 17, Dentatorubral Pallidoluysian Atrophy (DRPLA), and Spinal and Bulbar Muscular Atrophy (SBMA).
